# Local Defence System in Healthy Lungs

**DOI:** 10.3390/clinpract11040088

**Published:** 2021-10-01

**Authors:** Elizabeta Lohova, Zane Vitenberga-Verza, Dzintra Kazoka, Mara Pilmane

**Affiliations:** Institute of Anatomy and Anthropology, Riga Stradins University, LV-1010 Riga, Latvia; Zane.Vitenberga-Verza@rsu.lv (Z.V.-V.); Dzintra.Kazoka@rsu.lv (D.K.); Mara.Pilmane@rsu.lv (M.P.)

**Keywords:** healthy lungs, factors, immunity, respiratory system, local defence

## Abstract

Background: The respiratory system is one of the main entrance gates for infection. The aim of this work was to compare the appearance of specific mucosal pro-inflammatory and common anti-microbial defence factors in healthy lung tissue, from an ontogenetic point of view. Materials and methods: Healthy lung tissues were collected from 15 patients (three females and 12 males) in the age range from 18 to 86. Immunohistochemistry to human β defensin 2 (HBD-2), human β defensin 3 (HBD-3), human β defensin 4 (HBD-4), cathelicidine (LL-37) and interleukine 17A (IL-17A) were performed. Results: The lung tissue material contained bronchial and lung parenchyma material in which no histological changes, connected with the inflammatory process, were detected. During the study, various statistically significant differences were detected in immunoreactive expression between different factors in all lung tissue structures. Conclusion: All healthy lung structures, but especially the cartilage, alveolar epithelium and the alveolar macrophages, are the main locations for the baseline synthesis of antimicrobial proteins and IL-17A. Cartilage shows high functional plasticity of this structure, including significant antimicrobial activity and participation in local lung protection response. Interrelated changes between antimicrobial proteins in different tissue confirm baseline synergistical cooperation of all these factors in healthy lung host defence.

## 1. Introduction

The respiratory system is one of the main entrance gates for infection, so it is important for the organism to complement it with several innate host defence components. It is necessary to protect the organism from pathogen penetration, that is why the respiratory system is equipped with different protection mechanisms. Lungs have a huge surface and at the same time the alveolar epithelium is very thin and delicate, in order to be able to provide the exchange of oxygen and carbon dioxide [1]. Such a structure increases risk of developing an infection in the lungs and spreading the infection throughout the whole organism. Intact epithelium with mucociliary clearance, antimicrobial activity of the airway surface fluid, opsonization with surfactant protein and bactericidal activity of the alveolar macrophages are necessary to prevent penetration of particles and infection agents through the upper respiratory system and to prevent induction of inflammation in the lungs [2]. Mutual cooperation of different immune factors allows for stronger immune response and protection of the organism against pathogens.

Antimicrobial peptides (AMPs) comprise a large family of effector molecules of the innate immune defence. Two families of AMPs, defensins and cathelicidines, are the main antimicrobial molecules in the respiratory system defence [3]. The main attention in antimicrobial activity in the respiratory system is attracted by such members of human beta defensin family as human beta defensin 2 (HBD-2), human beta defensin 3 (HBD-3) and human beta defensin 4 (HBD-4). The cathelicidine family includes only one human cathelicidine—LL-37—whereby antimicrobial activity is one of its many functions.

Defensins are small, cationic antimicrobial polypeptides and take part in immune host defence, acting as innate and adaptive immunity mediators against bacteria, fungi, viruses and parasites [4]. HBD-2 is encoded by defensin beta 4 (DEFB4) gene, which is stimulated by a wide spectrum of immune cells, such as monocytes, macrophages, granulocytes and so on, but the main source of the HBD-2 is the epithelium [5,6,7]. Relevant studies demonstrated that tumour necrosis factor α (TNF-α), interleukin 1β (IL-1β), IL-22, extracellular Ca^2+^ and lipopolysaccharides (LPS) are elicited in HBD-2 mRNA induction through Toll-Like receptors (TLRs), dependent phosphoinositide 3-kinase (PI3K), nuclear factor-kappa B (NFkB) and mitogen-activated protein kinase (MAPK) pathways [4,8,9,10,11]. It demonstrates the large number of possibilities for mobilizing HBD-2, supporting the theory of its importance and superiority as an antimicrobial peptide. It is important to mention that HBD-2 is expressed in low doses in healthy lung tissues and is retained in surfactant to protect the respiratory system by direct destruction of pathogens or synergistic work with other antimicrobial molecules (lactoferrin) [12]. It is interesting to note that Gram-negative bacteria-induced HBD-2 mobilization is higher compared to the same situation with Gram-positive bacteria. It can be concluded that HBD-2 is able to fully destroy *Pseudomonas aeruginosa*, but not *Staphylococcus aureus* [13]. Main action of HBD-2 is broad spectrum antimicrobial activity mainly with anti-inflammatory activity. HBD-2 has the ability to suppress inflammatory process in lungs by decreasing IL-9, IL-13, IL-1β, IL-6 and TNF-α and, also, HBD-2 increases anti-inflammatory mediator IL-10 expression and improves pulmonary function [14,15]. HBD-2 chemoattracting abilities induce dendritic cell and T cell migration through C-C chemokine receptor type 6 (CCR6) [16]. Varied ways of HBD-2 activation, HBD-2 high expression in the epithelium and other tissues, variable functions and high activity in host defence prove antimicrobial peptide importance in respiratory host defence.

HBD-3 is encoded by DEFB103 gene, and its activity has been detected primarily in epithelial cells of the respiratory tract and genitourinary tract [17,18]. Induction of the HBD-3 gene is mediated by TNF-α, interferon γ (INF-γ), IL-1, lipopolysaccharides (LPS) and other factors, through specific Toll-Like receptors (TLR2/5/9) that leads to NFkB and MAPK pathway activation [19]. As all defensins, HBD-3 has pro- and anti-inflammatory activity. In contrast to HBD-2, HBD-3 shows significant activity towards *Staphylococcus aureus* and it is a more potent antimicrobial peptide against Gram-positive bacteria in the respiratory tract [18]. Furthermore, salt sensitivity, which was observed in HBD-2 and HBD-4, has not been detected in HBD-3 [20], this demonstrates the increased ability of the mediator to adapt and remain active during significant environmental changes. HBD-3, the same as HBD-2, can mobilize dendritic cells and memory T cells trough CCR6 [21]. Furthermore, it has been detected that it stimulates monocyte migration and activation trough CCR2 for macrophage recruitment [22].

HBD-4 are small cationic peptides whose expression was observed in epithelial cells and neutrophils [23]. Despite HBD-4 high expression in the epithelium of the testis and the gastric antrum, HBD-4 was also detected in lungs, but in a smaller amount [24]. HBD-4 gene expression is upregulated by endogenous (TNF-α, IL-1β, Ca^2+^ and phorbol 12-myristate 13-acetate (PMA)) and exogenous (LPS) factors [25]. It is important to mention, that HBD-4 activity against *Pseudomonas aeruginosa* is stronger than the activity of any other β-defensin [24]. HBD-4 has direct antimicrobial activity and is involved in cytokine and chemokine production such as IL-10, IL-6, INF-γ, IP-10 and others [22]. Interesting to mention that collaboration of HBD-4 with other antimicrobial peptides like HBD-3 and lysozyme was observed that induce mast cell migration, degranulation, prostaglandin D2 (PGD2) generation and chemotaxis [26,27]. HBD-4 has been identified in cartilage and showed weak expression in healthy tissues [28].

Every defensin has its own dominant function in host defence, but their fulfilling activity of each other (HBD-3 activity against Gram-negative bacteria is stronger than HBD-2 activity) and synergistic behaviour (HBD-3 and HBD-4 together stimulate mast cell migration) provide the highest antimicrobial activity.

Human cathelicidine (LL-37) is encoded by the cathelicidine antimicrobial peptide (CAMP) gene and has a broad-spectrum of antimicrobial activity against Gram-positive and Gram-negative bacteria, viruses, fungi and protozoa [29,30]. LL-37 is produced by mucosal epithelial cells, keratinocytes, adipocytes and immune cells [1,31]. Furthermore, it has been observed that vitamin D or calcitriol significantly induces expression of cathelicidine mRNA and synthesis of intracellular human cationic antimicrobial peptide (hCAP-18) pro-peptide form [32]. The expression of hCAP-18 and its transformation to the antimicrobial active form is induced by proinflammatory cytokines, growth factors, by other endogenous factors, and by exogenous factors like LPS [31,33]. LL-37 has two major functions in host defence. The first one is direct inhibition of pathogens. LL-37 recruit neutrophils, monocytes and T cells by connecting to formyl peptide receptor-like 1 (FPRL-1) and glyceraldehyde 3-phosphate dehydrogenase (GAPDH) [1,34]. The second one is modulation of innate and adaptive immune responses. LL-37 activate macrophages through epidermal growth factor receptor (EGFR) and inhibit their response to LPS [1,34]. LL-37 anti-inflammatory activity is expressed as IL-1β, IL-6, IL-8 and TNF-α production reduction through TLR2 and TLR4 [35].

Although, antimicrobial peptides are the main members of respiratory system immune defence, some other mediators are involved in stimulation of AMPs secretion and even in synergistic cooperation with these antimicrobial peptides. One of these mediators is interleukin 17A (IL-17A), which is involved in antimicrobial peptide gene regulation and cooperation with them.

IL-17A is a member of the big proinflammatory mediator family and it is important for host defence at mucosal surfaces. The main source of IL-17 was detected to be CD4+ T helpers, which became known as “Th17 cells”, but its production is also mediated by other immune cells such as natural killer cells, γδ-T cells, type 3 innate lymphoid cells (ILC3), neutrophils and macrophages [36,37,38]. Its powerful induction of inflammation is due to synergistical activity with other stimuli by stabilization of target mRNA [20,39]. During the acute lung inflammatory process, IL-17A induced gene stabilization and mediator production and lead to the mobilization of phagocytes and production of antimicrobial peptides [37,40]. One of these peptides is HBD-2, whose expression is regulated by IL-17A through JAK/NFkB pathway [41]. Interestingly, LL-37 also induces IL-17A expression. Moreover, long activation of IL-17A by LL-37 and cooperation of these two mediators, prolong and enhance the main inflammatory processes in tissues [42]. Overall, the synergy of LL-37 and IL-17A, HBD-3 and HBD-4 supports the hypothesis that synergistic cooperation of different immune factors can lead to a higher response to pathogens and faster reduction of inflammation.

According to the above-mentioned, the aim of this work was to compare the appearance of specific mucosal pro-inflammatory and common anti-microbial defence factors in healthy lung tissue, from an ontogenetic point of view.

## 2. Materials and Methods

### 2.1. Material Characteristics of Subjects

This study was approved by the Ethics Committee for Clinical Research of Medicine and Pharmaceutical Products at Pauls Stradins Clinical University Hospital Development Foundation in Latvia (Nr. 230113-17L, 2013). The study was conducted at the Institute of Anatomy and Anthropology, Latvia. All of the patients gave their informed written consent to participate in the study. The respiratory system material was obtained from 15 patients, 12 of them being males in the age range from 19–86 years and 3 of them females in the age range from 18 to 59 years. Tissues were not associated with inflammation or any other pathology. The material was collected during postmortem autopsy from persons who died in an accident and not from respiratory tract disease or during lung operation. Tissue material was collected from Pauls Stradins Clinical University hospital and Riga Stradins University Anatomy and Anthropology institute archive (lung tissue material) collection. The autopsy material contained bronchial wall tissue including cartilage from main and lobar bronchi, and lung parenchymal tissue. The lung tissue material used in the study was obtained at autopsy 12–24 h after the biological death of patients.

For patient selection, to exclude as many co-factors and confounders as possible, inclusion and exclusion criteria were developed.

Inclusion criteria: (1) A patient older than seven years (age when the lungs are considered morphologically mature and corresponding to the lung morphology of an adult individuals); (2) the obtained lung tissue sample histologically complies with the requirements of the tissue sample determined in the study and contains bronchial material (and/or lung parenchymal material).

Exclusion criteria: (1) Pathological finding in the lung tissue material (inflammatory cell infiltration, chronic inflammation, etc.); (2) acute or chronic lung disease in medical history; (3) lung oncological disease; (4) no bronchial and/or pulmonary material is found in the tissue section. The causes of death were accidents or fatal self-harm (trauma to body parts and organs, suicides that were not compatible with life). The causes of death of 6 individuals were associated with acute cardiovascular failure and/or ischemic heart disease (cause of death—cardiac arrest).

### 2.2. Immunohistochemical Analysis

The tissue specimens were fixed in a mixture of 2% formaldehyde and 0.2% picric acid in 0.1 M phosphate buffer (pH 7.2). Afterwards, they were rinsed in Tyrode buffer containing 10% saccharose for 12 h, then embedded into paraffin and cut into 3–4 μm thin sections. Biotin-Streptavidin biochemical method was used for immunohistochemistry (IMH) to detect: LL-37 (orb88370, working dilution 1:100, Biorbyt Limited, Cambridge, UK); HBD-4 (ab14419, working dilution 1:200, Abcam, San Francisco, CA, USA); HBD-3 (rb183268, working dilution 1:100, Biorbyt Limited, Cambridge, UK); HBD-2 (sc-20798, working dilution 1:100, Santa Cruz Biotechnology, Inc., Dallas, TX, USA); IL-17A (orb48920, working dilution 1:200, Biorbyt Limited, Cambridge, UK).

The stained slides were analysed by light microscopy (Leica RM2245, Leica Biosystems Richmond Inc., Richmond, Buffalo Grove, IL, USA) using nonparametric evaluation. The results were evaluated by grading the appearance of the positively stained cells in the visual field [43]. The designation was as follows: 0—no positive structures in the visual field; 0/+—occasional positive structures in the visual field; +—a few positive structures; +/++—a few to a moderate number of positive structures in the visual field; ++—a moderate number of positive structures in the visual field; ++/+++—moderate to numerous positive structures in the visual field; +++—numerous positive structures with the visual field; +++/++++—numerous to abundant positive structures in the visual field; ++++—abundant positive structures in the visual field.

For visual illustration, a Leica DM500RB digital camera (Leica Biosystems Richmond Inc., Richmond, Buffalo Grove, IL, USA) and Microsoft Photo editor (version 2021.21070.22007.0, Microsoft Corporation, Redmond, WA, USA) were used.

### 2.3. Statistical Analysis

Statistical data were ranked as ordinal values, where no positive structures (0) in the visual field were ranked with the value of 0, occasional positive structures (0/+) in the visual field were ranked with the value of 1, few positive structures (+) were ranked with the value of 2, a few to a moderate number of positive structures (+/++) were ranked with the value of 3, a moderate number of positive structures (++) in the visual field were ranked with the value of 4, moderate to numerous positive structures (++/+++) were ranked with the value of 5, numerous positive structures (+++) were ranked with the value of 6, numerous to abundant positive structures (+++/++++) were ranked with the value of 7 and abundant positive structures (++++) were ranked with the value of 8.

We used the non-parametric Wilcoxon test to compare the different mediator expression in the same structure and Spearman’s rank correlation coefficient (ρ), where ρ = 0–0.19 was assumed as a very weak correlation, ρ = 0.2–0.39 was assumed as a weak correlation, ρ = 0.4–0.59 was assumed as a moderate correlation, ρ = 0.6–0.79 was assumed as a strong correlation and ρ = 0.8–1 was assumed as a very strong correlation.

The statistical data processing was performed with IBM SPSS (Statistical Package for the Social Sciences) version 26.0 (IBM company, North Castle, Armonk, NY, USA). The significance level for all tests was selected as a *p*-value < 0.05 (5%).

## 3. Results

### 3.1. Tissue Review

The lung tissue material contained bronchial and lung parenchyma material in which no histological changes connected with inflammatory process were detected.

### 3.2. Immunohistochemical (IMH) Data

**Figure 1:**
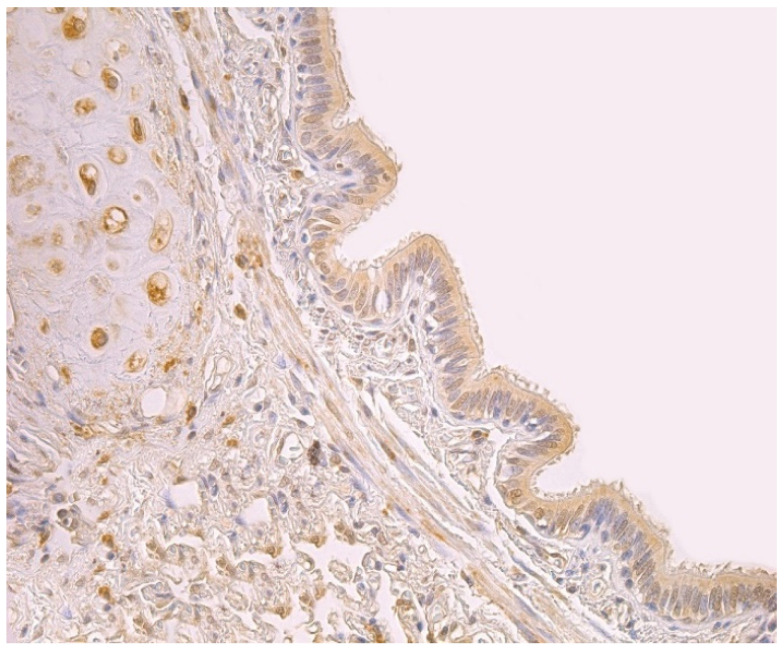
Note a moderate number of HBD-2 positive cells in the bronchial epithelium and cartilage of 59-year-old female bronchial wall. HBD-2 IMH, ×250.

The highest expression of HBD-2 was observed in cartilage where mainly a moderate number of positive structures was detected (Table 1, Figure 1). HBD-2 was present in a few to moderate number of alveolar macrophages and alveolar epithelium (Table 1), A few positive structures of HBD-2 appeared in connective tissue and bronchial epithelium (Table 1, Figure 1). Glands demonstrated only occasional number of positive HBD-2 cells (Table 1).

**Figure 2:**
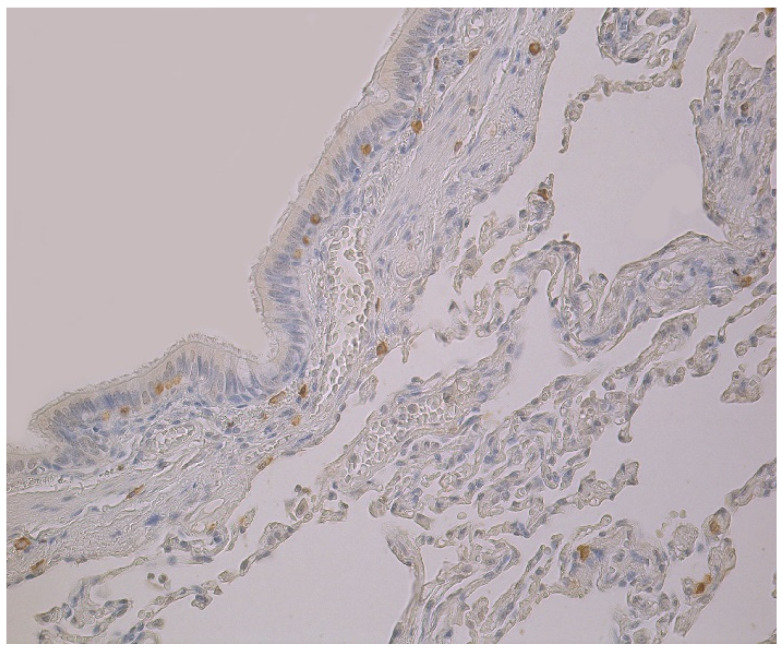
Note a few HBD-3 positive cells in the bronchial and alveolar epithelium, and in connective tissue of 59-year-old female bronchial wall. HBD-3 IMH, ×200.

Upon viewing HBD-3 in cartilage and alveolar epithelium, a moderate number of factor positive structures were detected in these locations (Table 1, Figure 2). A few to moderate HBD-3 positive structures were detected in alveolar macrophages (Table 1). Furthermore, glands, connective tissue and bronchial epithelium demonstrated only few positive cells (Table 1, Figure 2).

**Figure 3:**
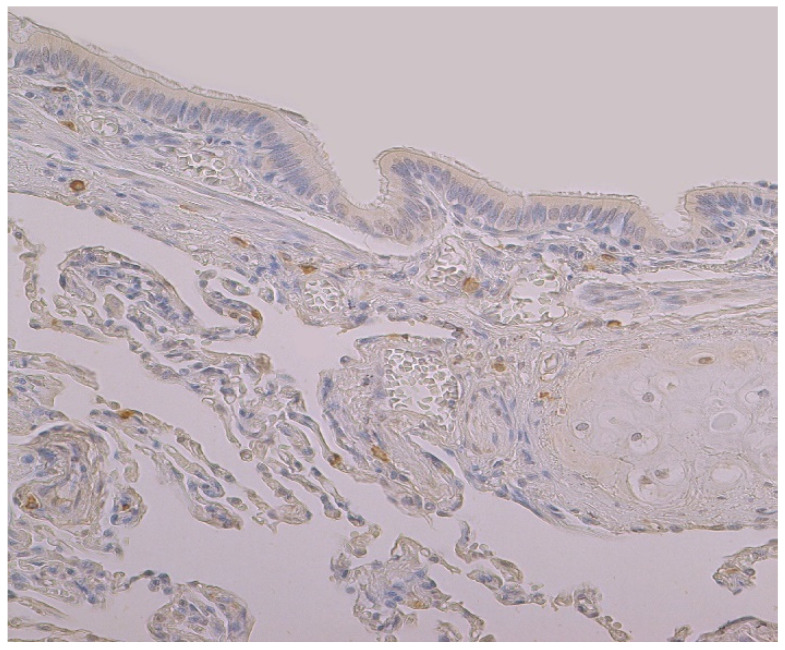
Immunohistochemical micrograph of occasional to few numbers of HBD-4 positive cells in connective tissue, cartilage and alveolar epithelium, but not in the bronchial epithelium of 74-year-old female. HBD-4 IMH, ×200.

The highest expression of HBD-4 was observed in cartilage of bronchial wall (moderate) (Table 2, Figure 3). A few to moderate HBD-4 expression was detected in alveolar epithelium and alveolar macrophages (Table 2, Figure 3). Occasional expression of HBD-4 was detected in connective tissue, glands and bronchial epithelium (Table 2, Figure 3).

**Figure 4:**
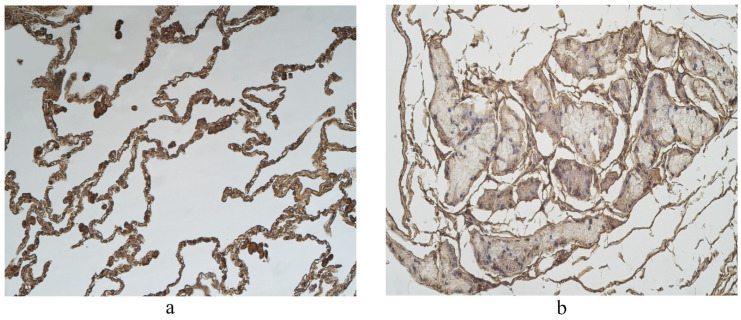
Immunohistochemical micrographs in lung tissue samples. (**a**) Note moderate number of IL-17A immunoreactive cells in the alveolar epithelium and numerous alveolar macrophages of 56-year-old male. IL-17A IMH, ×200; (**b**) a few IL-17A positive structures are observed in glands of 25-year-old male. IL-17A IMH, ×250.

**Figure 5:**
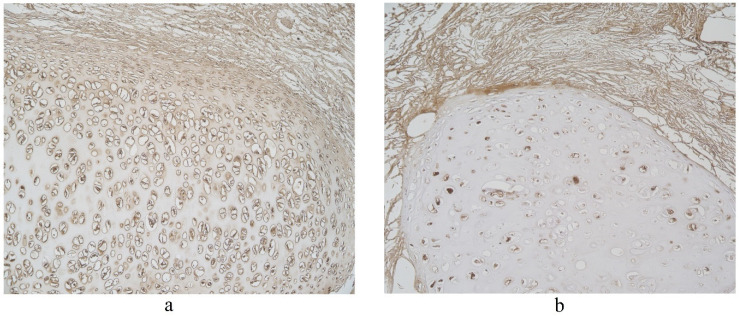
Immunohistochemical micrographs in lung tissue samples. (**a**) Numerous IL-17A positive cells in cartilage of 19-year-old male. IL-17A IMH, ×100; (**b**) moderate IL-17A positive structures in cartilage and none in connective tissue of 25-year-old male. IL-17A IMH, ×100.

**Figure 6:**
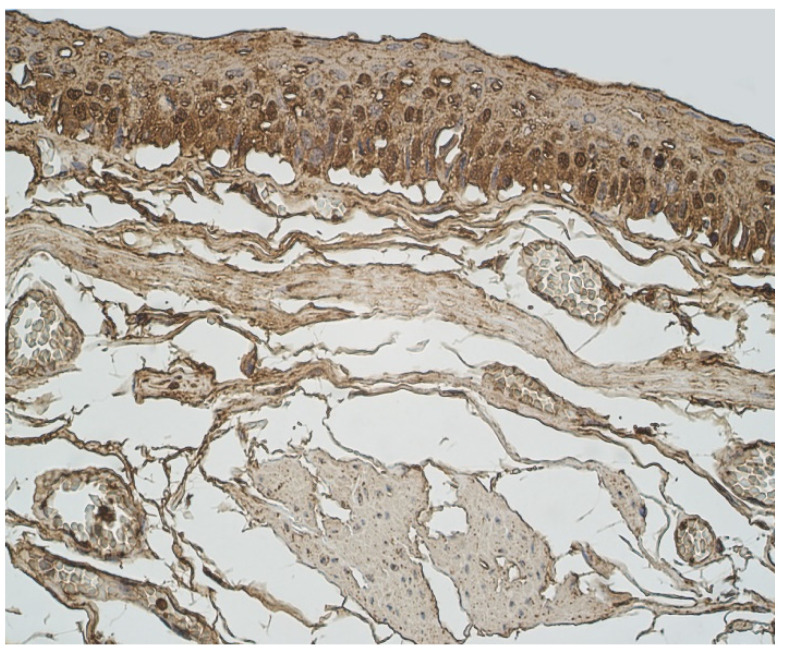
Immunohistochemical micrograph in lung tissue samples. Moderate number of IL-17A positive epitheliocytes and occasional number of positive connective tissue cells in 45-year-old male. IL-17A IMH, ×400.

The highest expression of IL-17A was observed in cartilage, where its expression fluctuated from moderate to numerous positive structures (Table 3, Figure 4a,b). A moderate number of IL-17A positive structures was detected in bronchial epithelium, alveolar epithelium and macrophages (Table 3, Figure 5a and Figure 6). A few IL-17A immunoreactive cells were detected in glands, but an occasional number of positive structures was detected in connective tissue (Table 3, Figure 5b).

**Figure 7:**
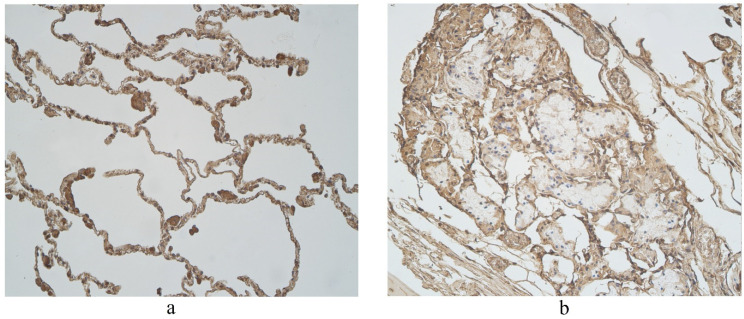
Immunohistochemical micrographs in lung tissue samples. (**a**) Few to moderate LL-37 immunoreactive cells in the alveolar epithelium and moderate to numerous LL-37 immunoreactive alveolar macrophages of 56-year-old male. LL-37 IMH, X200; (**b**) A few to moderate LL-37 positive structures in glands of 19-year-old male. LL-37 IMH, ×200.

**Figure 8:**
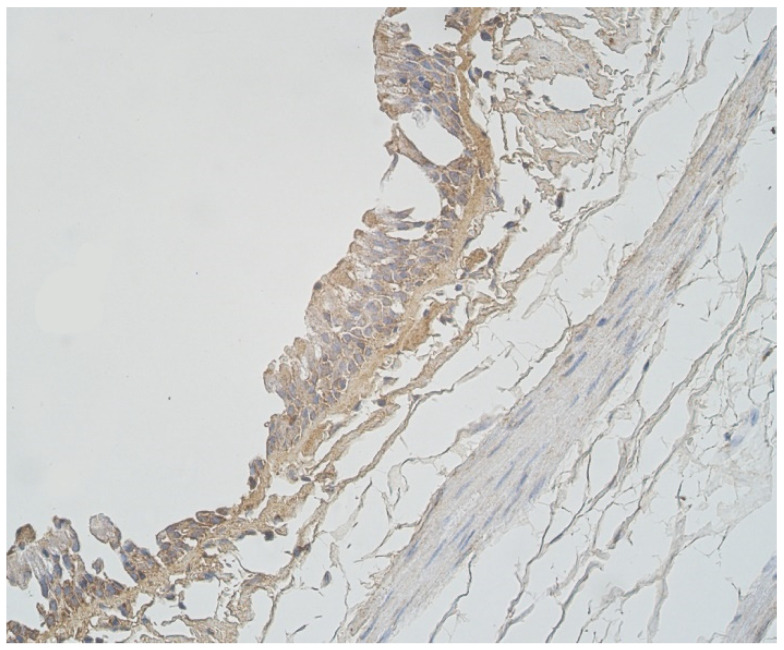
Note a few LL-37 positive epitheliocytes and occasional cathelicidine positive connective tissue cells of 56-year-old male. LL-37 IMH, ×250.

**Figure 9:**
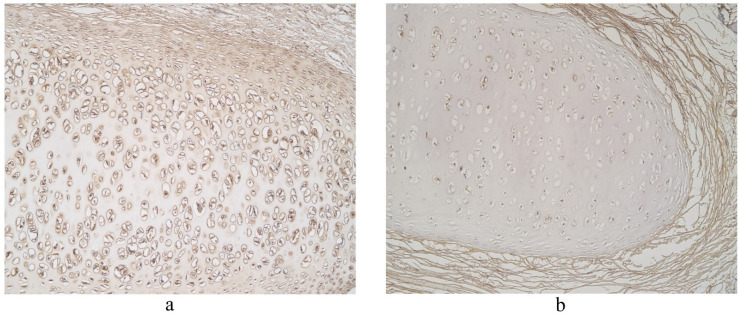
Immunohistochemical micrographs in lung tissue samples. (**a**) Note numerous LL-37 immunoreactive cells in cartilage of 19-year-old male. LL-37 IMH, X100; (**b**) A few LL-37 positive structures in cartilage of 19-year-old male. LL-37 IMH, ×100.

A moderate number of cathelicidine-positive structures was detected in bronchial cartilage, alveolar epithelium and alveolar macrophages (Table 3, Figure 7a and Figure 8). LL-37 was present in a few to moderate immunoreactive cells in bronchial epithelium (Table 3, Figure 9). Connective tissue and glands demonstrated only a few positive structures (Table 3, Figure 7b).

### 3.3. Statistical Analysis

**Figure 10:**
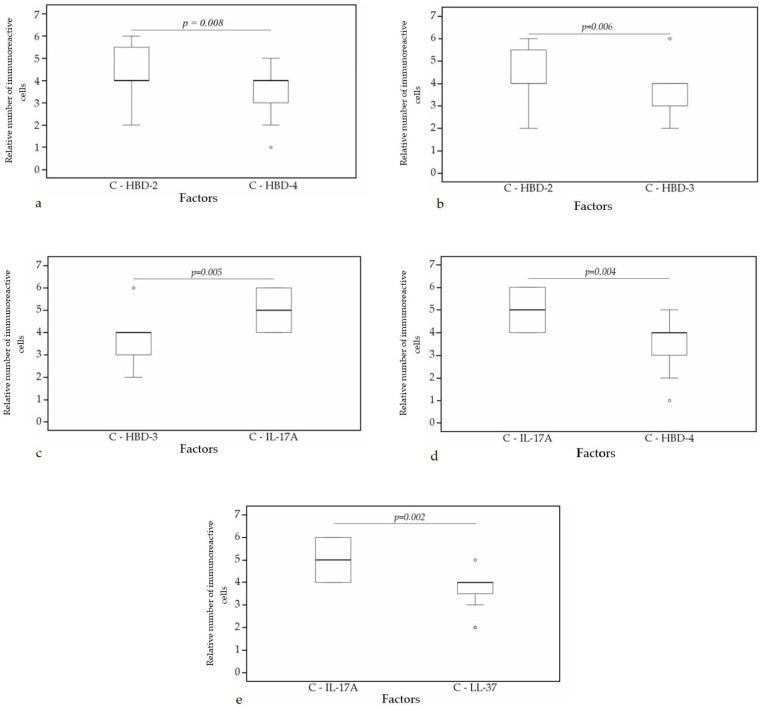
Wilcoxon test revealing statistically significant difference between the relative numbers of different factors in cartilage. (**a**) A statistically significant difference between HBD-2 and HBD-4 in cartilage. (**b**) A statistically significant difference between HBD-2 and HBD-3 in cartilage. (**c**) A statistically significant difference between HBD-3 and IL-17A in cartilage. (**d**) A statistically significant difference between IL-17A and HBD-4 in cartilage. (**e**) A statistically significant difference between IL-17A and LL-37 in cartilage. C, cartilage; HBD-2, human β defensin 2; HBD-3, human β defensin 3; HBD-4, human β defensin 4; LL-37, cathelicidine; IL-17A, interleukin 17A; o—outlier data—indicates a higher or lower relative number of positive cells which lie more than 1.5 but less than 3.0 times the interquartile range below the first quartile or above the third quartile.

**Figure 11:**
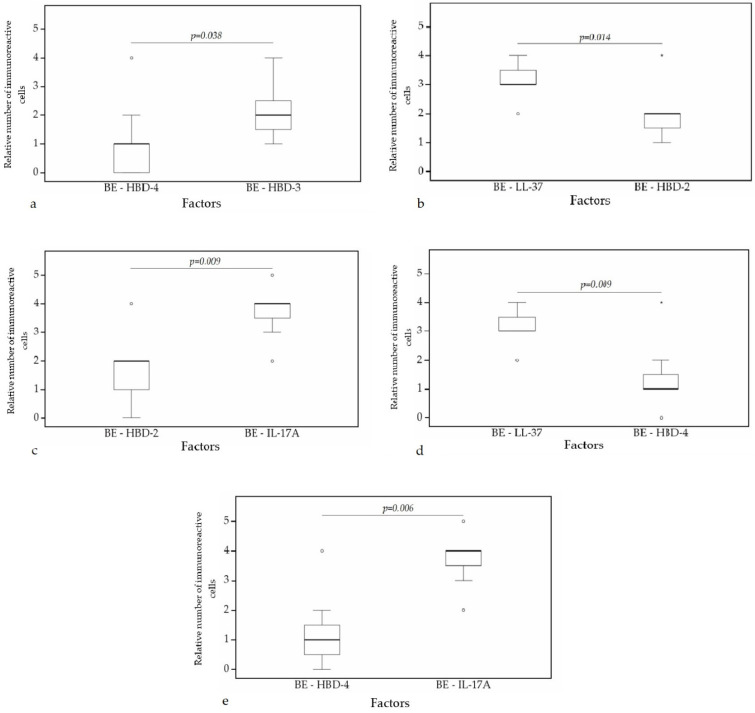
Wilcoxon test revealing statistically significant difference between the relative numbers of different factors in bronchial epithelium. (**a**) A statistically significant difference between HBD-4 and HBD-3 in bronchial epithelium. (**b**) A statistically significant difference between HBD-2 and LL-37 in bronchial epithelium. (**c**) A statistically significant difference between HBD-2 and IL-17A in bronchial epithelium. (**d**) A statistically significant difference between LL-37 and HBD-4 in bronchial epithelium. (**e**) A statistically significant difference between HBD-4 and IL-17A in bronchial epithelium. BE, bronchial epithelium; HBD-2, human β defensin 2; HBD-3, human β defensin 3; HBD-4, human β defensin 4; LL-37, cathelicidine; IL-17A, interleukin 17A; o—outlier data—indicates a higher or lower number of relative positive cells which lie more than 1.5 but less than 3.0 times the interquartile range below the first quartile or above the third quartile; *—extreme outlier data—indicates a higher or lower number of relative positive cells which lie more than 3.0 times the interquartile range below the first quartile or above the third quartile.

A statistically significant difference was found between HBD-4 and HBD-2 (*p* = 0.008) (Figure 10a), HBD-3 and HBD-2 (*p* = 0.006) (Figure 10b), HBD-3 and IL-17A (*p* = 0.005) (Figure 10c), HBD-4 and IL-17A (*p* = 0.004) (Figure 10d), IL-17A and LL-37 (*p* = 0.002) (Figure 10e) in cartilage. Furthermore, a statistical significance in bronchial epithelium was found between HBD-4 and HBD-3 (*p* = 0.038) (Figure 11a), HBD-2 and LL-37 (*p* = 0.014) (Figure 11b), HBD-2 and IL-17A (*p* = 0.009) (Figure 11c), HBD-4 and LL-37 (*p* = 0.009) (Figure 11d), HBD-4 and IL-17A (*p* = 0.006) (Figure 11e).

**Figure 12:**
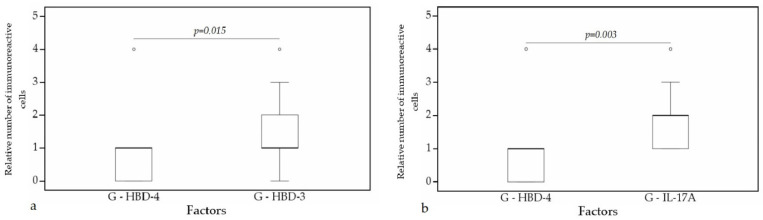
Wilcoxon test revealing statistically significant difference between the relative numbers of different factors in glands. (**a**) A statistically significant difference between HBD-4 and HBD-3 in glands. (**b**) A statistically significant difference between HBD-2 and LL-37 in glands. G, glands; HBD-3, human β defensin 3; HBD-4, human β defensin 4; IL-17A, interleukin 17A; o—outlier data—indicates a higher or lower number of relative positive cells which lie more than 1.5 but less than 3.0 times the interquartile range below the first quartile or above the third quartile.

**Figure 13:**
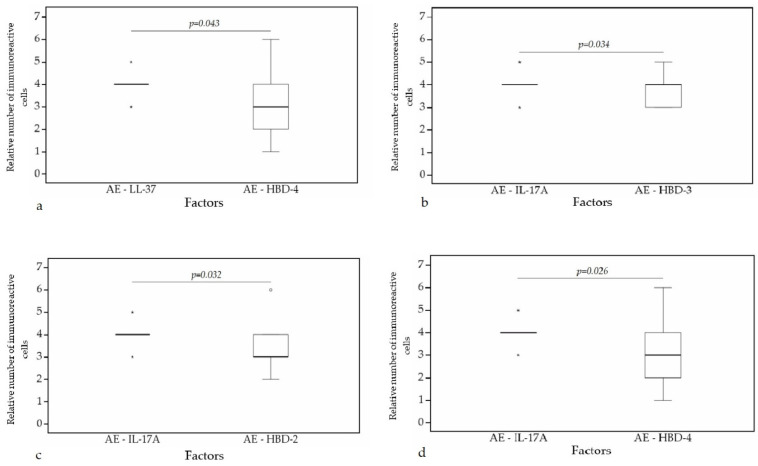
Wilcoxon test revealing statistically significant difference between the relative numbers of different factors in alveolar epithelium. (**a**) A statistically significant difference between LL-37 and HBD-4 in alveolar epithelium. (**b**) A statistically significant difference between IL-17A and HBD-3 in alveolar epithelium. (**c**) A statistically significant difference between IL-17A and HBD-2 in alveolar epithelium. (**d**) A statistically significant difference between IL-17A and HBD-4 in alveolar epithelium. AE, alveolar epithelium; HBD-2, human β defensin 2; HBD-3, human β defensin 3; HBD-4, human β defensin 4; LL-37, cathelicidine; IL-17A, interleukin 17A; o—outlier data—indicates a higher or lower number of relative positive cells which lie more than 1.5 but less than 3.0 times the interquartile range below the first quartile or above the third quartile; *—extreme outlier data—indicates a higher or lower number of relative positive cells which lie more than 3.0 times the interquartile range below the first quartile or above the third quartile.

**Figure 14:**
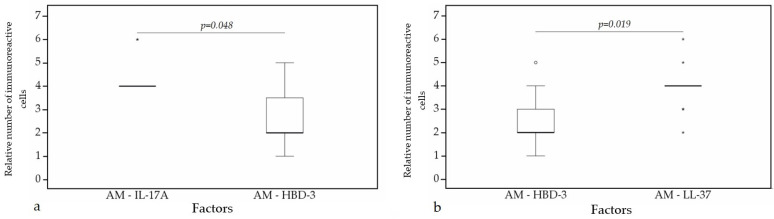
Wilcoxon test revealing statistically significant difference between the relative numbers of different factors in alveolar macrophages. (**a**) A statistically significant difference between IL-17A and HBD-3 in alveolar macrophages. (**b**) A statistically significant difference between HBD-3 and LL-37 in alveolar macrophages. AM, alveolar macrophages; HBD-3, human β defensin 3; LL-37, cathelicidine; IL-17A, interleukin 17A; o—outlier data—indicates a higher or lower number of relative positive cells which lie more than 1.5 but less than 3.0 times the interquartile range below the first quartile or above the third quartile; *—extreme outlier data—indicates a higher or lower number of relative positive cells which lie more than 3.0 times the interquartile range below the first quartile or above the third quartile.

A statistically significant difference was found between HBD-4 and HBD-3 (*p* = 0.015) (Figure 12a), HBD-4 and IL-17A (*p* = 0.003) (Figure 12b) in glands. Moreover, a statistically significant difference in alveolar epithelium was observed between HBD-4 and LL-37 (*p* = 0.043) (Figure 13a), HBD-3 and IL-17A (*p* = 0.034) (Figure 13b), HBD-2 and IL-17A (*p* = 0.032) (Figure 13c), HBD-4 and IL-17A (*p* = 0.026) (Figure 13d). HBD-3 and IL-17A (*p* = 0.048) (Figure 14a), HBD-3 and LL-37 (*p* = 0.019) (Figure 14b) demonstrated a statistically significant difference in alveolar macrophages.

A correlation analysis of the normal lung tissue demonstrated very strong positive correlation between HBD-3 in alveolar epithelium and HBD-3 in alveolar macrophages (ρ = 0.911, *p* < 0.001), HBD-2 and HBD-3 in alveolar epithelium (ρ = 0.845, *p* < 0.001), HBD-2 in alveolar epithelium and HBD-3 in alveolar macrophages (ρ = 0.838, *p* < 0.001) (Table 4).

Strong positive correlation was found between HBD-2 and HBD-4 in bronchial epithelium (ρ = 0.781, *p* = 0.001), HBD-3 in glands and bronchial epithelium (ρ = 0.733, *p* = 0.002), HBD-3 in glands and HBD-4 in bronchial epithelium (ρ = 0.729. *p* = 0.002), HBD-2 in alveolar epithelium and HBD-4 in alveolar macrophages (ρ = 0.707, *p* = 0.003), LL-37 and IL-17A in bronchial epithelium (ρ = 0.706, *p* = 0.003), IL-17A in glands and HBD-2 in cartilage (ρ = 0.705, *p* = 0.003), HBD-4 in alveolar epithelium and alveolar macrophages (ρ = 0.692, *p* = 0.004), HBD-3 and HBD-4 in bronchial epithelium (ρ = 0.685, *p* = 0.005), LL-37 and IL-17A in alveolar epithelium (ρ = 0.681, *p* = 0.005) (Table 4). In addition, strong positive correlation was detected between HBD-2 and HBD-3 in cartilage (ρ = 0.674, *p* = 0.006), IL17A and HBD-3 in alveolar epithelium (ρ = 0.670, *p* = 0.006), IL-17A and HBD-2 in alveolar epithelium (ρ = 0.659, *p* = 0.008), LL-37 and HBD-4 in alveolar macrophages (ρ = 0.658, *p* = 0.008), IL-17A in alveolar epithelium and HBD-3 in alveolar macrophages (ρ = 0.645, *p* = 0.009), LL-37 in bronchial epithelium and HBD-3 in glands (ρ = 0.640, *p* = 0.010), LL-37 in alveolar epithelium and HBD-3 in alveolar macrophages (ρ = 0.639, *p* = 0.010), LL-37 and HBD-2 in bronchial epithelium (ρ = 0.630, *p* = 0.012), IL-17A and HBD-4 in alveolar epithelium (ρ = 0.628, *p* = 0.012) (Table 4).

## 4. Discussion

Our results generally covered healthy lungs, particularly the cartilage, alveolar epithelium and the alveolar macrophages as the main places for the antimicrobial proteins and IL-17A synthesis as the local defence factors here were observed in moderate number of cells in all the patients.

AMPs and IL-17A production is carried out mainly by epithelial and immune cells. Relevant studies have demonstrated dominant AMPs and IL-17A expression by alveolar epithelium and alveolar macrophages during early infection, and showed necessity for lower pathogen agent concentration to stimulate expression of these factors in the alveolar epithelium and alveolar macrophages than in other structures of the respiratory system [44,45,46]. That shows importance of the alveolar epithelium and alveolar macrophages in the innate immune response of the lungs. An unexpected finding was AMP, especially LL-37 and IL-17A, high expression in hyaline cartilage of the lungs. Expression of these factors in cartilage most likely demonstrate plasticity of the structure, its importance in local tissue defence and its ability in rapid adaptation. Many different factors are involved in the development, adaptation and regeneration of cartilage, the role of which at first seems clear. Vascular endothelial growing factor (VEGF) and fibroblast growing factor 18 (FGF18) are involved in fetal cartilage development and in processes associated with cartilage damage. Although both factors’ roles in cartilage development are similar, relevant studies demonstrated VEGF degradational effect; however, in contrast, FGF18 protective and regeneration stimulating effect on cartilage in osteoarthritis [47,48,49]. Our knowledge of cartilage, especially in the respiratory system (tracheal and bronchial cartilages), its secreted factors and its role in our organism is limited and requires more in-depth research.

According to our data, the bronchial epithelium with relatively indistinct expression of antimicrobial proteins, and exceptionally expressing the LL-37 and IL-17A in moderate number of cells, probably indicates this airway epithelial barrier as more plastic under the direct different influences of the environment.

Expression of LL-37 as an inactive pro-peptide and intracellular collection of factors occurs not only in immune cells such as neutrophiles and macrophages, but also in epithelial cells of the respiratory system, especially after vitamin D stimulation [31]. These reserves of the factor can be easily mobilized with minimal concentration of air containing exogenous triggers [33]. What is interesting, is the wide spectrum of stimuli which can provoke LL-37 expression and activation—Gram-positive and Gram-negative bacteria, viruses, fungi and protozoa. Activated LL-37 induces dose dependent IL-17A production which in turn provide other antimicrobial peptide gene stabilization and expression [37]. The cooperation of these two mediators provides first line defence against pathogens. Although HBD-2 is considered as the most highly expressed defensin in respiratory system, its expression in the bronchial epithelium is lower than in the alveolar epithelium, and HBD-2 secretion is induced mainly by Gram-negative bacteria [12]. HBD-3 and HBD-4 expression in the respiratory system is not as high as LL-37 and HBD-2, but their activity against different pathogens like Gram-positive and Gram-negative bacteria, and HBD-3 lack of sensitivity to salt concentration changes, demonstrate these factors as an additional protective step in innate immunity [18,20,24]. Based on the above mentioned and our gathered data, we suggest that the uneven expression of AMPs and IL-17A in the bronchial epithelium shows a multi-level system of immune defence and plasticity of the epithelial barrier of the respiratory system against various trigger factors.

Immunohistochemical reaction demonstrated related changes of HBD-3 and HBD-4 expression in different tissues that seemingly are associated with synergistic cooperation of these two mediators in the lung host defence. This idea is confirmed by the strong positive correlation between HBD-3 and HBD-4 in glands, bronchial and alveolar epithelium. Relevant studies showed that HBD-3 is a more potent mediator against Gram-positive *Staphylococcus aureus*, but HBD-4 demonstrated its full dominance as AMP against Gram-negative *Pseudomonas aeruginosa* [18,24]. HBD-3 and HBD-4 direct antimicrobial activity is different, and mediators complement each other to provide better protection against pathogens that invade the respiratory system. According to research of Xuejun Chen et al., the involvement of both mediators is important to ensure adequate mast cells mobilization: HBD-3 demonstrated its dominance in mast cells degranulation and PGD2 generation, but HBD-4 promoted more enhanced migration of mast cells than HBD-3 [27]. Interaction of HBD-3 and HBD-4 in mast cells activation and migration shows synergistic involvement of mediators in inflammatory reaction moderation. The above-mentioned leads to the conclusion that the synergistic co-working of these two mediators provide highly potent antimicrobial activity in different respiratory system tissues based on the innate immune response.

HBD-2 secretion in healthy bronchial cartilage of our subjects was statistically significant compared with HBD-3 and HBD-4. According to other studies, expression of HBD-2 was not seen in healthy articular cartilage, but, after tissue stimulation with IL-1β, IL-6, TNF-α and Pseudomonas aeruginosa, HBD-2 mRNA induction was detected [50,51]. Such opposing activity of the HBD-2 in cartilage can be explained by tissue location and intensity of different trigger factor influences. Each day our respiratory system filtrates approximately 11,000 litres of the air which contains macro- and microscopical particles, including bioaerosols (dead and alive fungi and bacteria, bacterial endotoxins, mycotoxins, pollen and so on), peeled skin and dust [52]. Bioaerosols induce IL-1β IL-6, TNF-α and other pro-inflammatory factor production [53,54], which activate HBD-2 mRNA through different pathways (PI3K, NFkB, MAPK) leading to antimicrobial and anti-inflammatory reactions and immune system homeostasis.

Interestingly, HBD-2 role in cartilage is not only associated with antimicrobial activity and anti-inflammatory effects, but it is also involved in cartilage repair and cell proliferation. Relevant studies have indicated HBD-2 role as a link between antimicrobial activity and cartilage remodelling processes by involvement in proliferation and differentiation of chondrocytes [50,55]. This demonstrates multifunctional activity of HBD-2 in the human organism.

An interesting finding was increased secretion of LL-37 and IL-17A in cartilage, mainly in the proliferation zone. LL-37 and IL-17A are mainly produced by epitheliocytes and immune cells and are involved in inflammatory process regulation. Relevant studies demonstrated LL-37 and IL-17A involvement in some inflammatory processes of joints, like inflammatory arthritis, where both of these mediators stimulate significant TNFα secretion and development of chronic inflammation, but immune cells and synovial cells were the source of LL-37 and IL-17A in inflammatory arthritis, not hyalinocytes [42,56]. Moreover, we have not found any mention of cathelicidine and IL-17A secretion in the cartilage. As a result, this is the first detection of LL-37 and IL-17A expression in hyaline cartilage. Both of these mediators are inflammatory process regulators, which shows their possible involvement in lung cartilage immune processes, but more detailed research should be conducted to determine the specific role of IL-17A and LL-37 in hyaline cartilage of the lungs.

LL-37 and IL-17A have demonstrated interaction with each other, which was shown in our research by the intercorrelation between LL-37 and IL-17A in the bronchial epithelium, connective tissue and cartilage. Other studies have indicated that LL-37 alone can selectively regulate inflammation-related processes through the NF-kB signalling pathway [57]. This regulation proceeds by increased phosphorylation of IkappaB (IkB) kinase (IKK) and p65 [42]. Meanwhile, IL-17A alone also stimulates proinflammatory processes through NF-kB and boosts the phosphorylation of p65 and IkB and slightly increases that of IKK [42,58]. Chakkrapong Kuensaen demonstrated IL-17A early expression after stimulation with LL-37 and higher gene expression response to combined stimulation with both mediators than LL-37 alone [42]. As a result, LL-37 activates and cooperates with IL-17A, leading to inflammation intensification and prolongation long term.

## 5. Conclusions

Regardless of age, all healthy lung structures, but especially the cartilage, alveolar epithelium and the alveolar macrophages, are the main locations for the baseline synthesis of antimicrobial proteins and IL-17A, while the bronchial epithelium (as a structure more sensitive to the influence of environment) produces only indistinct baseline antimicrobial protein levels with exception for IL-17A and cathelicidine to be expressed in numerous cells.

Significant detection of all antimicrobial proteins in healthy lung hard tissue—cartilage—shows high functional plasticity of this structure, including significant antimicrobial activity and participation in local lung protection response.

Interrelated changes between antimicrobial proteins HBD-3, HBD-2, HBD-4, LL-37 and IL17A, but especially between HBD-3 and HBD-4 expression in different tissues, confirms baseline synergistic cooperation of all these factors in the healthy lung host defence, regardless of age.

## Figures and Tables

**Table 1:** Relative number of HBD-2 and HBD-3-positive structures in the healthy lung structures: cartilage, connective tissue, glands, bronchial epithelium, alveolar macrophages and alveolar epithelium.

Nr	Age	HBD-2						HBD-3					
		C	CT	G	BE	AE	AM	C	CT	G	BE	AE	AM
1	18	+++	+	+	+	0	0	++	+	+/++	+/++	0	0
2	19	+++	+/++	+/++	++	0	0	+/++	+	++	+	0	0
3	19	++	0	0	+	+	+	++	+	0/+	0/+	+/++	+
4	25	+++	+/++	++	0	+/++	+/++	+++	+/++	+	++	+/++	+
5	27	++	++	+	0/+	+/++	++	++	0/+	+	+	++	++
6	29	++/+++	0/+	0/+	0	+++	++	++	0/+	0/+	0	++/+++	++/+++
7	33	++	++	0/+	0	++	+	++	0/+	0	0	++	++
8	35	+/++	+/++	0/+	0	+	+	+	+/++	0/+	0/+	+/++	0/+
9	38	++/+++	0/+	0	0/+	+/++	++	++	0	+	+/++	+/++	+
10	45	++	0	0/+	+	++	++	+/++	+	+	+	++	+/++
11	55	+	+	0	0	+/++	++/+++	+	0/+	0/+	+	++	+/++
12	56	++	+/++	0/+	+	+/++	++	+/++	+	0/+	+	+/++	0/+
13	59	+++	+	0	0/+	+/++	0/+	++	0	0	0/+	++	+
14	74	++	0	+	0	++	++	+/++	0/+	0/+	0	++	+/++
15	86	+/++	0/+	0/+	0	+/++	++	+/++	0	0/+	0	+/++	+
	Common	++	+	0/+	+	+/++	+/++	++	+	+	+	++	+/++

Abbreviations: C, cartilage; CT, connective tissue, G, glands; BE, bronchial epithelium; AE, alveolar epithelium; AM, alveolar macrophages; HBD-2, human β defensin 2; HBD-3, human β defensin 3; 0—no positive structures in the visual field; 0/+—occasional positive structures in the visual field; +—few positive structures; +/++—few to moderate number of positive structures in the visual field; ++—moderate number of positive structures in the visual field; ++/+++—moderate to numerous positive structures in the visual field; +++—numerous positive structures with the visual field.

**Table 2:** Relative number of HBD-4-positive structures in the healthy lung structures: cartilage, connective tissue, glands, bronchial epithelium, alveolar macrophages and alveolar epithelium.

Nr	Age	HBD-4					
		C	CT	G	BE	AE	AM
1	18	+/++	0	0/+	+	0	0
2	19	++	+	++	++	0	0
3	19	+/++	0/+	0/+	0/+	+	+
4	25	++/+++	++	0/+	0/+	+++	++
5	27	++	0/+	0	0	+/++	0/+
6	29	++	+	0/+	0	++	+++
7	33	+/++	0/+	0	0	++	++
8	35	+/++	0	0	0	+/++	+
9	38	++	+	0/+	0/+	++	++
10	45	0/+	+	0	0/+	+	+++
11	55	+/++	0/+	0	0	+/++	++
12	56	++	0/+	0/+	0/+	+/++	++
13	59	++	0	0	0	0/+	0/+
14	74	++	0/+	0/+	0	+/++	+
15	86	+	0/+	0/+	0	+	0/+
	Common	++	0/+	0/+	0/+	+/++	+/++

Abbreviations: C, cartilage; CT, connective tissue, G, glands; BE, bronchial epithelium; AE, alveolar epithelium; AM, alveolar macrophages; HBD-4, human β defensin 4; 0—no positive structures in the visual field; 0/+—occasional positive structures in the visual field; +—few positive structures; +/++—few to moderate number of positive structures in the visual field; ++—moderate number of positive structures in the visual field; ++/+++—moderate to numerous positive structures in the visual field; +++—numerous positive structures with the visual field.

**Table 3:** Relative number of cathelicidine and IL-17A-positive structures in the healthy lung structures: cartilage, connective tissue, glands, bronchial epithelium, alveolar macrophages and alveolar epithelium.

Nr	Age	LL-37						IL-17A					
		C	CT	G	BE	AE	AM	C	CT	G	BE	AE	AM
1	18	++	+	+	+/++	0	0	+++	0/+	+/++	+/++	0	0
2	19	++/+++	+	+	++	0	0	+++	+	++	++/+++	0	0
3	19	++	0/+	0	+	++_v_	++	++/+++	+	+	+	+/++	++
4	25	++	0/+	0/+	0	++	++/+++	++	0	+	0	++	++
5	27	++	+	+	+/++	++	+/++	+++	+	+	++	++	++
6	29	++	0/+	+	+	++	++	+++	0	+	+	++_v_	++_v_
7	33	++	0/+	+	0	++/+++	++	++/+++	0/+	+	0	++/+++	++
8	35	+/++	+	0/+	0	++	++	++_v_	0/+	0/+	0	++_v_	+++
9	38	+/++	0/+	0/+	+/++	+/++	+/++	++	0	+	++	++_v_	++
10	45	++	0	+	++	++_v_	++	++/+++	0/+	0/+	++	++_v_	++_v_
11	55	++_v_	+	0/+	0	++	++	++	+	+	++	++/+++	++
12	56	+	0/+	0/+	+/++	++	+++	++	0/+	0/+	++	++	+++
13	59	+	0/+	0/+	0	+/++	++	++/+++	0/+	+/++	0	++_v_	++
14	74	++	+	+	+/++	++_v_	++_v_	++	+	0/+	++	++_v_	0
15	86	++	0/+	0/+	+/++	++	+	+++	0	0/+	0	++	++_v_
	Common	++	+	+	+/++	++	++	++/+++	0/+	+	++	++	++

Abbreviations: C, cartilage; CT, connective tissue, G, glands; BE, bronchial epithelium; AE, alveolar epithelium; AM, alveolar macrophages; LL-37, cathelicidine; IL-17A, interleukin 17A; 0—no positive structures in the visual field; 0/+—occasional positive structures in the visual field; +—few positive structures; +/++—few to moderate number of positive structures in the visual field; ++—moderate number of positive structures in the visual field; ++/+++—moderate to numerous positive structures in the visual field; +++—numerous positive structures with the visual field.

**Table 4:** Spearman’s rank correlation coefficient revealed correlations between the relative numbers of different factors in healthy lung tissue.

Strength of Correlation	Marker 1	Marker 2	Rho (ρ)	*p*-Value
Very strong positive correlation	HBD-3 in alveolar epithelium	HBD-3 in alveolar macrophages	0.911	<0.001
	HBD-2 in alveolar epithelium	HBD-3 in alveolar epithelium	0.845	<0.001
	HBD-2 in alveolar epithelium	HBD-3 in alveolar macrophages	0.838	<0.001
Strong positive correlation	HBD-2 in bronchial epithelium	HBD-4 in bronchial epithelium	0.781	0.001
	HBD-3 in glands	HBD-3 in bronchial epithelium	0.733	0.002
	HBD3 in glands	HBD-4 in bronchial epithelium	0.729	0.002
	HBD-2 in alveolar epithelium	HBD-4 in alveolar macrophages	0.707	0.003
	LL-37 in bronchial epithelium	IL-17A in bronchial epithelium	0.706	0.003
	IL-17A in glands	HBD-2 in cartilage	0.705	0.003
	HBD-4 in alveolar macrophages	HBD-4 in alveolar epithelium	0.692	0.004
	HBD-3 in bronchial epithelium	HBD-4 in bronchial epithelium	0.685	0.005
	LL-37 in alveolar epithelium	IL-17A in alveolar epithelium	0.681	0.005
	HBD-2 in cartilage	HBD-3 in cartilage	0.674	0.006
	IL-17A in alveolar epithelium	HBD-3 in alveolar epithelium	0.670	0.006
	IL-17A in alveolar epithelium	HBD-2 in alveolar epithelium	0.659	0.008
	LL-37 in alveolar macrophages	HBD-4 in alveolar macrophages	0.658	0.008
	IL-17A in alveolar epithelium	HBD-3 in alveolar macrophages	0.645	0.009
	LL-37 in bronchial epithelium	HBD-3 in glands	0.640	0.010
	LL-37 in alveolar epithelium	HBD-3 in alveolar macrophages	0.639	0.010
	LL-37 in bronchial epithelium	HBD-2 in bronchial epithelium	0.630	0.012
	IL-17A in alveolar epithelium	HBD-4 in alveolar epithelium	0.628	0.012

Abbreviations: HBD-2, human β defensin 2; HBD-3, human β defensin 3; HBD-4, human β defensin 4; LL-37, cathelicidine; IL-17A, interleukin 17A.
